# Context Based Predictive Information

**DOI:** 10.3390/e21070645

**Published:** 2019-06-29

**Authors:** Yuval Shalev, Irad Ben-Gal

**Affiliations:** Laboratory for AI, Machine Learning, Business & Data Analytics, Department of Industrial Engineering, The Tel-Aviv University, Ramat-Aviv 6997801, Israel

**Keywords:** context tree, predictive information, time series analysis, information bottleneck

## Abstract

We propose a new algorithm called the context-based predictive information (CBPI) for estimating the predictive information (PI) between time series, by utilizing a lossy compression algorithm. The advantage of this approach over existing methods resides in the case of sparse predictive information (SPI) conditions, where the ratio between the number of informative sequences to uninformative sequences is small. It is shown that the CBPI achieves a better PI estimation than benchmark methods by ignoring uninformative sequences while improving explainability by identifying the informative sequences. We also provide an implementation of the CBPI algorithm on a real dataset of large banks’ stock prices in the U.S. In the last part of this paper, we show how the CBPI algorithm is related to the well-known information bottleneck in its deterministic version.

## 1. Introduction

Shannon’s mutual information (MI) [1] is a widely-used measure in various research domains. The MI between two random variables evaluates in bits the reduction of the uncertainty (entropy) of one of the variables given the other (i.e., averaged over all possible realizations of the other). This measure is non-parametric and hence can be used to describe complex interactions. Predictive information (PI) [2] was proposed as a measure of MI between one time series’ past and another’s future (either the same one or a different time series). One example is the MI between different stocks’ prices over different time periods, as discussed next. If we limit the prediction to one step ahead, the PI is directly related to the known transfer entropy (TE) [3,4] that measures the conditional PI between input and output time series, given the past realizations of the output. For the past two decades, TE has been widely used for analyzing information flow between different time series and causality analysis (see a comprehensive review on TE and related measures at [4,5]). However, a difficulty arises when these measures need to be estimated from data. The number of possible sequences for which information contributions should be estimated is exponential in the number of time lags. In some cases, when most realized past sequences are uninformative about the future, false information contributors lead to overestimation of PI, hence associating predictive power with uninformative sequences. We call this condition sparse PI (SPI).

When dealing with real valued data, one can assume an AR-based model as in [5,6] to estimate PI with relatively low complexity and high accuracy. Nevertheless, this assumption often fails when the times lags between the series are not linearly correlated, or in the discrete data case. Methods that rely on non-parametric approaches are at risk of encountering a large estimation error under the SPI conditions. Such methods are based on commonly-used MI estimation methods ranging from naive binning to bias corrections and nearest neighbor methods (see for example the benchmark study in [7]). When applied to time series, these methods resolve the time dimensionality challenge by keeping relevant time lags entirely, even if some of them contain few informative sequences, and so, positive bias occurs. Thus, these methods do not apply estimation correction at a realization level, which removes a significant share of uninformative sequences, yet enables an improved explainability at the realization level.

We provide a solution for estimating the PI in discrete data, which is based on an expansion of the lossy compression context tree (CT) algorithm [8,9,10], which is called the input/output context tree (I/O CT) [11,12].

This algorithm parses the input time series into a tree of contexts (sequences), where in each node, the conditional probability of the output given the context is assigned. Only nodes with conditional probabilities that are significantly different from those of their parent nodes (this difference is often measured by the Kullback–Leibler distance) are kept, and the others are pruned. The pruning stage is governed by the pruning constant c [9,10]. In the SPI condition, c has a significant impact even for relatively small values, where most of the nodes are pruned. On the other hand, c should not be too high, to avoid pruning of informative nodes. Usually, c is determined heuristically or by cross-validation.

The CT (and I/O CT) algorithm, as well as other algorithms from the variable order Markov models family were proposed to overcome overfitting in learning tasks such as classification and prediction [11]. Estimating the information between a time series’ past and future was usually not one of the tasks for which these algorithms were used.

In this paper, we show how to estimate PI between time series as the sum of the Kullback–Leibler divergence [13] between the root node and the leaves of I/O CT. Furthermore, we utilize the dependency of PI on the pruning constant to determine the value of c that enables good separation among informative and uninformative contexts. This is done without the need for an additional run over the data as in, e.g., cross-validation procedures.

The proposed procedure is implemented by a new context-based predictive information (CBPI) algorithm: First, a full I/O CT is built (c = 0). Second, the PI is calculated for ascending values of c. Third, by identifying the threshold at which redundant information is removed, a value of c is chosen to return an estimation for the “filtered” PI and most of the informative sequences.

The CBPI algorithm is designated for discrete data. Nevertheless, it can be implemented also on real valued data by performing any type of binning, such as described in [7]. In many cases, binning results in some loss of information [7,14], due to the compression procedure. Often the number of bins controls such loss, where a large number of bins leads to a closer distribution to the real valued data and vice versa. An important consideration is the relevancy of the binning to the domain from which data are sourced. The approach taken in this paper, following other works (e.g., [15,16,17]), is that after removing the uninformative sequences, domain-relevant insights could be immediately drawn. We also note that methods that are applied directly on real valued data are limited when dealing with time series where non-linear sequential characteristics are important (as opposed to treating every time lag as a feature on its own), and in addition, they suffer from limited explainability capabilities.

A relevant question to ask is whether SPI exists in practice. The generic answer is that it exists whenever compression of one time series with respect to another results in a significant reduction in data, while most important information is preserved. As an example, we can consider time series in a large stock market. Due to market efficiency, PI between time series of stock prices is expected to be small or equal to zero. In the case of a small PI, most of the past sequences are uninformative, and the SPI condition might occur (e.g., see [15] and the discussion therein). We discuss these types of scenarios in the Results Section and exemplify the CBPI algorithm’s contribution by implementing it on a simulated setup for benchmarking it relative to other methods, as well as on real stock market data of eight large banks in the U.S. In the latter example, we also demonstrate how the outcome of the CBPI algorithm can be exploited to gain important insights by performing a higher resolution analysis of the PI contributors.

In the last part of this paper, we discuss an interesting observation about the connection between the CBPI algorithm and the deterministic information bottleneck (DIB) [18]. The DIB is a variant of the well-known IB algorithm [19] that enables deterministic compression of one variable with respect to another. We give an intuitive explanation of this connection and formalize an optimization problem inspired by DIB that relies on the I/O CT algorithm. With the extraction of PI, I/O CT can serve as a complement to DIB in deterministic compression tasks of one time series with respect to another.

To conclude, the first main contribution of this paper is demonstrating the extraction of PI from an I/O CT constructed from input and output time series. The second contribution is introducing a novel algorithm, called the CBPI, for PI estimation, while offering a new method of identifying the value of the pruning constant that governs the compression rate. The third contribution is showing how the CBPI algorithm can be used for in-depth analysis of interaction fundamentals. Finally, the linkage of the context tree algorithm and the deterministic information bottleneck method is discussed by formalizing an optimization problem that describes the I/O CT algorithm.

This paper is organized as follows. Related work is reviewed in Section 2. Section 3 discusses basic concepts and the mathematical background of the ideas presented in this work. In Section 4, the extraction of PI from the I/O CT algorithm, the pruning constant’s identification, and the CBPI algorithm are described. Section 5 shows the experimental results of implementing CPBI in both simulated and real data settings. Section 6 discusses the general characteristics of the pruning constant and the relation between I/O CT and DIB. In Section 7, we conclude this work.

## 2. Related Work

MI was originally introduced by Shannon and discussed widely in the literature, with [1] being an extensively cited source. Many methods are used for MI estimation. The simplest and most straightforward one is the so-called plug-in method, where marginal and conditional probabilities are evaluated through counts [1]. Other existing methods of MI estimation, such as [20,21,22,23,24], use different corrections of probabilities due to the finite size of data. The K-nearest neighbors method proposed in [22] is one of the most commonly used in recent years.

PI was formulated by Bialek, Nemenman, and Tishby in [2]. There, the researchers discussed the theoretical attributes of PI and its connection to important aspects of model learning. Empirically measuring PI is important for the estimation of TE, which was initially proposed by [3]. To calculate TE, one should estimate a conditional PI, although this is not stated directly in papers that discuss TE.

Researchers such as [14,25] used standard methods of MI estimation, mentioned in the beginning of this section, to estimate TE. Those studies proposed algorithms to avoid dimensionality explosion due to the time dimension by removing uninformative time lags. According to those methods, when a specific time lag is found to be informative in a specific realization, all its realizations, including uninformative ones, are included in the estimation. In sequential data, where the number of different realizations is potentially large, this drawback can be crucial by adding many uninformative sequences to the estimation.

To overcome this challenge, we utilize the CT algorithm, a lossy compression member of the family of variable order Markov models that were originally constructed for compression of a single time series and found to be well-suited to the prediction task of discrete time series [9,15,26]. These  works extend the basic Markov model by proposing an algorithm for data compression that extract important sequences (i.e., “contexts”) not necessarily of the same order, which contained significant information about the conditioned symbol. The CT algorithm constructs a universal source. This means that the model can asymptotically replace any source from the class of tree sources with optimal code length, hence approaching the sequence’s entropy with the optimal convergence rate [9].

Variable order Markov models and their usage have been extensively explored, e.g., for prediction tasks [15,26,27], time series classification [28,29], clustering [30,31], anomaly detection [10,32], and modeling DNA sequences [11,33]. Two works were found that incorporated variable order models and information or entropy. In [21], the authors used several models from this family to estimate the entropy of a sequential source. In [28], the goal was to construct a discriminative model (to perform the classification task) for time series, with each series belonging to one of *K* classes. The MI between a symbol and a class k∈K given the contexts was obtained from the prediction suffix tree algorithm to find discriminative contexts (that the researchers called features).

Ben-Gal et al. [11] and later Brice et al. [12] proposed an input/output formulation of the context tree algorithm (I/O CT), where the branches of the context tree belong to one time series and the leaves belong to another. In this way, the researchers could incorporate data from different time series for learning tasks, such as structure learning and anomaly detection within the CT framework.

The pruning constant c is the only hyper-parameter in the I/O CT algorithm. When properly tuned, it reduces the variance of the model while keeping a low bias. In [9], c was suggested to be $\frac{1}{log(d)}$, where *d* is the alphabet size, without any clear theoretical justification. Model evaluation methods such as the AIC [34,35] were proposed in [36,37] in relation to pruning constant determination. They involve a brute force search for *c* by calculating the log-likelihood over the training data for each value of c. A structured method that provides justification for c based on data characteristics is yet to be proposed.

A relevant practical fact when considering large datasets is that the I/O CT algorithm can be distributed to multiple machines similarly to other algorithms from the same family [38,39,40]. In this paper, the key-value approach is utilized, where the keys are the contexts and the values are the counters of the output time series, similar to [38,40].

In the final part of this paper, we discuss the relation between I/O CT and the IB [19]. IB and its implications for understanding learning mechanism have been extensively explored (for example see [41]). In [42,43,44], PI was incorporated theoretically in the IB framework.

The deterministic IB (DIB) [18] is a variant of IB that leads to hard clustering, which we will refer to in the final part of this paper.

## 3. Preliminaries and Mathematical Background

Henceforth, unless stated otherwise, random variables are represented by uppercase letters, while their realizations are denoted by lowercase letters; multi-dimensional variables are denoted by bold letters and sets with calligraphic letters.

**Mutual information [1]:** Given two discrete random variables X and Y, the mutual information between them is defined as:

(1) $$\begin{array}{c} I\left(X;Y\right)=\sum_{x\in X}\sum_{y\in Y}P\left(x,y\right)log\frac{P(x,y)}{P(x)P(y)}. \end{array}$$

I(X;Y) is a positive symmetrical measure. The  **Kullback–Leibler divergence** ($D_{KL}$) between arbitrary probability functions Q(·) and P(·) is given by:

(2) $$\begin{array}{c} \begin{array}{c} D_{KL}\left(Q\left(X,Y\right)\|P\left(X,Y\right)\right)=\sum_{x\in X}\sum_{y\in Y}Q\left(x,y\right)log\frac{Q(x,y)}{P(x,y)}. \end{array} \end{array}$$

Following Equation (2), I(X;Y) can be written as:

(3) $$\begin{array}{c} I\left(X;Y\right)={\langle D_{KL}\left(P\left(Y|X\right)\|P\left(Y\right)\right)\rangle }_{P(X)}, \end{array}$$

where ${\langle \cdot \rangle }_{P(\cdot )}$ is the expectation with respect to the subscript distribution.

**Predictive information (PI) [44]:** The MI between two random vectors, one representing the past $\tau_{p}$ time lags, ${\overset{\leftarrow }{X}}_{\tau_{p}}$, and another representing time series values from the future $\tau_{f}$ time lags, ${\vec{X}}_{\tau_{f}}$, can be measured by the PI. Following Equation (3), PI can be defined by using $D_{KL}$:

(4) $$PI\left({\overset{\leftarrow }{X}}_{\tau_{p}};{\vec{X}}_{\tau_{f}}\right)={\langle D_{KL}\left(P\left({\vec{X}}_{\tau_{f}}|{\overset{\leftarrow }{X}}_{\tau_{p}}\right)\|P\left({\vec{X}}_{\tau_{f}}\right)\right)\rangle }_{P({\overset{\leftarrow }{X}}_{\tau_{p}})}.$$

**Context tree (CT) algorithm [9,10]:** Given a sequence of length *N*, $x^{N}$, generated from a tree source *X*, the CT algorithm finds a finite set S of size |S| of contexts $S(x^{N})$. S satisfies the requirement that the conditional probability to obtain a symbol given the whole sequence preceding that symbol is close enough to the conditional probability of obtaining the symbol given a context, i.e.,

(5) $$\begin{array}{c} P\left(x|x^{N}\right)≅P\left(x|S\left(x^{N}\right)\right). \end{array}$$

Given Equation (5), when |S| sequences are informative, the number of conditional probability parameters needed to describe $x^{N}$ equals |S|(d−1), where *d* is the alphabet size of *X*.

To obtain S, the learning algorithm constructs a context tree where each node holds a set of ordered counters that represent the distribution of symbols that follow that context, which is defined by the path to that node [10]. At the next step, a pruning procedure is performed to leave only those contexts in S (also called optimal contexts [10]), with corresponding nodes in the tree that represent the conditional distribution of the output variable conditioned on the context, which is different from the distributions of the output variable conditioned only on part of the context. This difference between the conditioned distributions is measured by the $D_{KL}$ measure. The pruning phase is regulated by the pruning constant c, which is a tunable hyper-parameter. As proven by [9], this procedure leads to the optimal description of the tree source, e.g., one with the minimal description length of the generating tree source. Descriptions of the main principles of the CT Algorithm, how to obtain S, and a numerical example appear in Appendix A.

The I/O CT [11,12] algorithm is a generalization of the CT algorithm where the tree’s contexts are from the input sequence and the leaves represent counters of the output sequence, in contrast to Equation (5), where the input and the output are from the same time series,

(6) $$\begin{array}{c} P\left(y|x^{N}\right)≅P\left(y|S\left(x^{N}\right)\right). \end{array}$$

**Figure 1:**
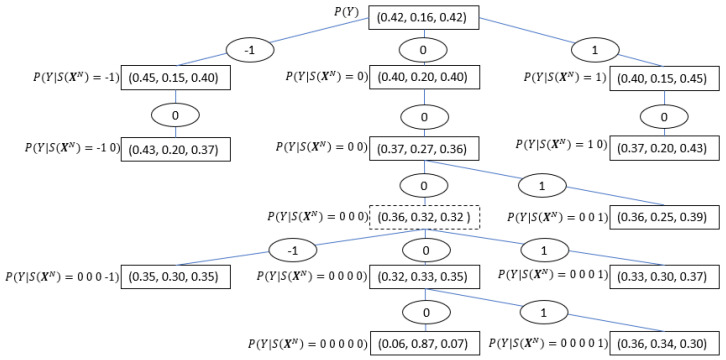
I/O CT based on Table 1 as obtained from HSBC (input) to Deutsche Bank (output) stock prices’ time series. Each edge represents a single realization. Consecutive edges represent contexts (sequences) in reverse order. The nodes represent the conditional probabilities of the output given the input context between the root to that node. The root (at the top of the tree) contains the marginal distribution of the output time series. The dashed node is the only one in this case that does not represent an optimal context.

Figure 1 along with Table 1, show an I/O CT example based on the results from the real data experiment discussed in Section 5.2, where the symbols -1, 0, 1 denote negative, zero and positive change respectively in the financial time series. By investigating the tree structure, one can extract important characteristics of the considered time series interaction, such as the maximal memory of the process (represented by the tree depth, which in this case equals five), the significant sequences, and their conditioned probabilities, which in this case are relatively symmetric with respect to a zero change, etc. See further discussion in the Results Section.

## 4. The Context-Based Predictive Information Algorithm

Let $X^{N}$ and $Y^{N}$ be the input and the output time series of length *N*, respectively. The PI of the input about the output can be stated similarly to Equation (4), where in this case, the past and the future trajectories belong to different time series. $\left\{\overset{\leftarrow }{x};\tilde{\overset{\leftarrow }{x}}\right\}\in {\overset{\leftarrow }{x}}_{\tau_{p}}$ represent the informative and uninformative contexts respectively from the input time series; $\vec{y}\in {\vec{y}}_{\tau_{f}}$ represents the sequences from the output time series; and $\hat{PI}\left({\overset{\leftarrow }{x}}_{\tau_{p}};{\vec{y}}_{\tau_{f}}\right)$ represent the estimated PI. We aim to remove $\tilde{\overset{\leftarrow }{x}}$, for which the true $D_{KL}\left(P\left(\vec{y}|\tilde{\overset{\leftarrow }{x}}\right)\|P\left(\vec{y}\right)\right)=0$, although the estimated $D_{KL}\left(\hat{P}\left(\vec{y}|\tilde{\overset{\leftarrow }{x}}\right)\right\|\hat{P}\left(\vec{y}\right)>0$, because this measure is positively biased due to the finite size of the data [7]. This will be done while keeping the informative sequences. In the SPI condition (see Section 1), where $\frac{|\overset{\leftarrow }{x}|}{|\tilde{\overset{\leftarrow }{x}}|}<<1$, removing these contexts can significantly decrease the $\hat{PI}$ estimation error. Namely, removing a large number of contexts with positively-biased information contribution that tend to zero when the data size is growing will reduce the total positive biased estimation error that comes from uninformative sequences.

To achieve this goal, we apply some of the principles implemented in [28] to introduce a novel method for $\hat{PI}$ estimation using the I/O CT. As shown in Figure 1 and discussed in Section 3, the root node of the I/O CT represents the marginal (unconditioned) distribution of $Y^{N}$ (the symbols’ frequency in $Y^{N}$). The estimated PI between the input and the output time series is the sum of the $D_{KL}$ between the probability in the root node and the conditional probability distributions given the contexts in S, weighted by the probabilities of these contexts, following Equation (4)

(7) $${\hat{PI}}_{c}=\langle D_{KL}(\hat{P}(\vec{y}|S_{c}\left(\overset{\leftarrow }{x}\right)\|\hat{P}\left(\vec{y}\right){)\rangle }_{\hat{P}\left(S_{c}\left(\overset{\leftarrow }{x}\right)\right)},$$

where ${\hat{PI}}_{c}$ is the empirical PI obtained from the I/O CT algorithm with a pruning constant c and $S_{c}\left(\overset{\leftarrow }{x}\right)$ is its corresponding optimal context set. To continue with the running example of banks’ data, we use Table 1, which represents the tree in Figure 1; using Equation (7), $\hat{PI}$ with c = 1 can be calculated as  follows:

(8) $$\begin{array}{c} \begin{array}{c} {\hat{PI}}_{1}=\\ 0.369\cdot D_{KL}\left(0.45,0.15,0.40\right)\left|\right|\left(0.42,0.16,0.42\right)+\\ 0.111\cdot D_{KL}\left(0.40,0.20,0.40\right)\left|\right|\left(0.42,0.16,0.42\right)+\\ \ldots +\\ 0.005\cdot D_{KL}\left(0.06,0.87,0.07\right)\left|\right|\left(0.42,0.16,0.42\right)=0.016\;\mathrm{bits}. \end{array} \end{array}$$

So far, the extraction of $\hat{PI}$ from CT with a given c value has been described. A tuning method for finding the value of c that provides a good separation between informative and uninformative contexts is now proposed by utilizing the statistics gained by the first stage in the CT algorithm. Consider the vector **c** of indexed pruning constant values $c_{i}$. The empirical second derivative of ${\hat{PI}}_{c_{i}}$ with respect to $|S_{c_{i}}|$ can be obtained by [45]:

(9) $$\begin{array}{c} \frac{\partial^{2}{\hat{PI}}_{c_{i}}}{\partial |S_{c_{i}}{|}^{2}}\approx \frac{{\hat{PI}}_{c_{i+1}}+{\hat{PI}}_{c_{i-1}}-2{\hat{PI}}_{c_{i}}}{\left(\right|S_{c_{i+1}}\left|-\right|S_{c_{i-1}}{\left|\right)}^{2}}. \end{array}$$

When the absolute value of (9) reaches a greater value than a threshold ϵ, the correspondent pruning constant is chosen. The second derivative is used to enable the detection of changes from higher than a linear order (e.g., curved-shaped changes) in the $\hat{PI}$. A linear decrease is expected to happen when uninformative contexts are removed. The reason for this behavior lies in the pruning threshold of the CT algorithm. This threshold equals to the probability of a context times a parent-child $D_{KL}$ measure. In the uninformative case, incrementally increasing the pruning constant will result in the pruning of all the leaves at the same tree level in the reverse order. Hence, in each incremental increase in the pruning constant c, the same size of $\hat{PI}$ is subtracted. When one of the contexts contains a significant amount of information, its pruning will result in a higher order change in the empirical PI.

$\hat{PI}$ extraction and tuning of the pruning constant c constitute the CBPI algorithm. Let us summarize the CBPI algorithm, which is described in Algorithm 1: First, a full CT is constructed (by setting the pruning constant to *c* = 0). Then, for an increasing order of the pruning constant value with a fixed step, a CT is constructed for each pruning constant, and its estimated PI is calculated. This  procedure repeats itself until the second derivative condition (which is exemplified by the curve in Figure 5) is satisfied and the algorithm stops and returns the values of the pruning constant c, as well as the PI of the last iteration.

Considering the $\hat{PI}$ randomness, we need to reject the null hypothesis that $\hat{PI}$ = 0, especially in the case of SPI condition. Here, we adopt the approach of [46] by setting the stopping threshold ϵ to be higher than the 95 percentile value of the $\hat{PI}$ obtained by repeatedly reshuffling the time series and measuring the resulting $\hat{PI}$.

**Algorithm 1** Context-based predictive information: pseudocode.
  1:**Input**: $x^{N}$, $y^{N}$, **c**, ϵ  2:Implement on $x^{N}$, $y^{N}$ the first stage of the I/O CT algorithm to obtain a full I/O context tree  3:**for***i* in 1 to |c|−1 **do**  4: Implement the following stages of the I/O CT algorithm  5: with $c_{i-1}$, $c_{i}$, $c_{i+1}$, and obtain $S_{c_{i-1}}$, $S_{c_{i}}$, $S_{c_{i+1}}$  6: Calculate ${\hat{PI}}_{c_{i-1}},{\hat{PI}}_{c_{i}},{\hat{PI}}_{c_{i+1}}$  7: **if** |$S_{c_{i-1}}$| = |$S_{c_{i+1}}$| **then**  8:  dv2←0  9: **else**10:  $dv2\leftarrow |\frac{\partial^{2}{\hat{PI}}_{c_{i}}}{\partial |S_{c_{i}}{|}^{2}}|$11: **end if**12: **if** dv2>ϵ **then**13:  return $c_{i}$, ${\hat{PI}}_{c_{i}}$14: **end if**15:
**end for**
16:return $c_{\left|c\right|},0$


### Complexity Analysis

The complexity of the CBPI is comprised of two main parts: (i) the complexity of the context tree construction, which is constructed in the first part of the algorithm; and (ii) the calculation of the PI in every iterative step at the second stage of the algorithm. The construction of the full I/O CT has a complexity of O(NlogN), where *N* is the input sequence size [47]. The complexity of the PI calculation depends linearly on the number of contexts in the tree, which is the sum of a geometric series of length $l_{max}$, the length of the longest context in the tree. In each one of |c| iterations, one needs at most $d\frac{1-d^{l_{max}}}{1-d}∼d^{l_{max}}$ summation operations, where *d* denotes the alphabet size. Accordingly, the full complexity of the CBPI algorithm is:

(10) $$\begin{array}{c} O(NlogN+|c|d^{l_{max}}). \end{array}$$

As mentioned in Section 1, the left complexity term can be controlled by a distributed tree construction procedure via multiple processors, while the right complexity term can be controlled by limiting the depth of the full context tree, which makes sense since the probability for extracting significant sequences decreases with the sequence length.

## 5. Empirical Results

This section shows the results of a simulation setup with a known ground truth, which is used to measure the performance of the CBPI algorithm compared to benchmark methods mentioned earlier. Later, a real financial dataset is used as an example of the CBPI algorithm’s usage for PI estimation and a high-resolution data analysis.

### 5.1. PI Estimation in SPI Conditions: A Simulated Study

In this example, $\hat{PI}$ is measured between an input time series with an alphabet size of d=10 and an output binary time series, both having 5000 symbols each. The SPI condition was generated by randomly selecting a small number of input contexts as conditioning random vectors, while assigning to each of these contexts a conditioned probability of the output time series’ symbols. Three hundred simulation runs were executed, where in each simulation run, the number of optimal contexts, the length of each context, and the conditioning probabilities were randomly generated. Then, a random source generated the time series according to these conditioning values. Table 2 summarizes the key features of this simulation setup. By knowing that the true probability of any of the contexts is $d^{-l}$ where *l* is the length of the context, we calculated the marginal distribution of every symbol, as well as its conditional probability given its context in the series. Given the optimal contexts, we could calculate the true PI value and compare it to the estimated ones that were obtained from the CBPI algorithm, as well as the benchmark methods in every simulation.

Table 3 shows the mean absolute difference between the true and estimated values, as well as the error with respect to the true value. This table shows that the CPBI-based error is significantly smaller than those of all other methods that range between 70–510 percent.

**Figure 2:**
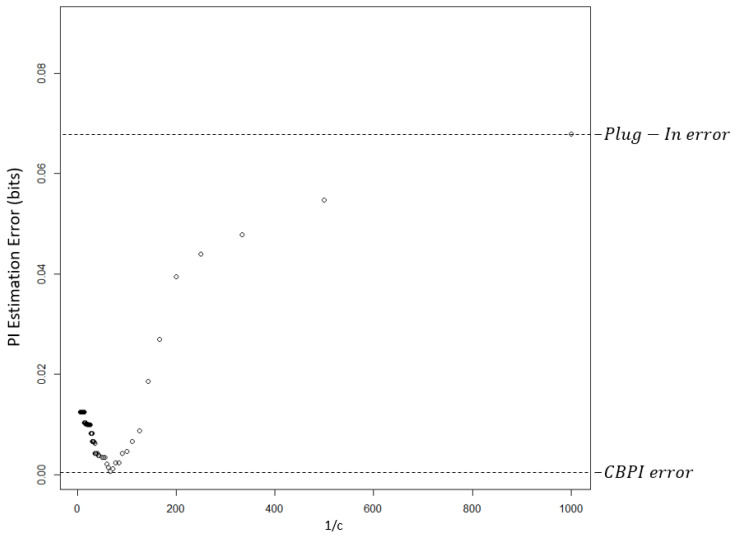
PI estimation absolute difference error as a function of $\frac{1}{c}$ in a single arbitrarily-chosen simulation run. The value of the pruning constant c that results in the lowest error is 0.017. The c value that was chosen by the CBPI algorithm was 0.02, with estimation error that was near to the minimum value. The error on the right edge was obtained with a full CT, an estimation that was equal to the one obtained by the plug-in method.

The dependency of the $\hat{PI}$ estimation error on c in a randomly-chosen single run is demonstrated in Figure 2. The smallest estimation error was obtained by the CBPI algorithm with a c value that corresponds to approximately $\frac{1}{c}=60$. The error at the right edge of this figure is equal to the error of the plug-in method.

**Figure 3:**
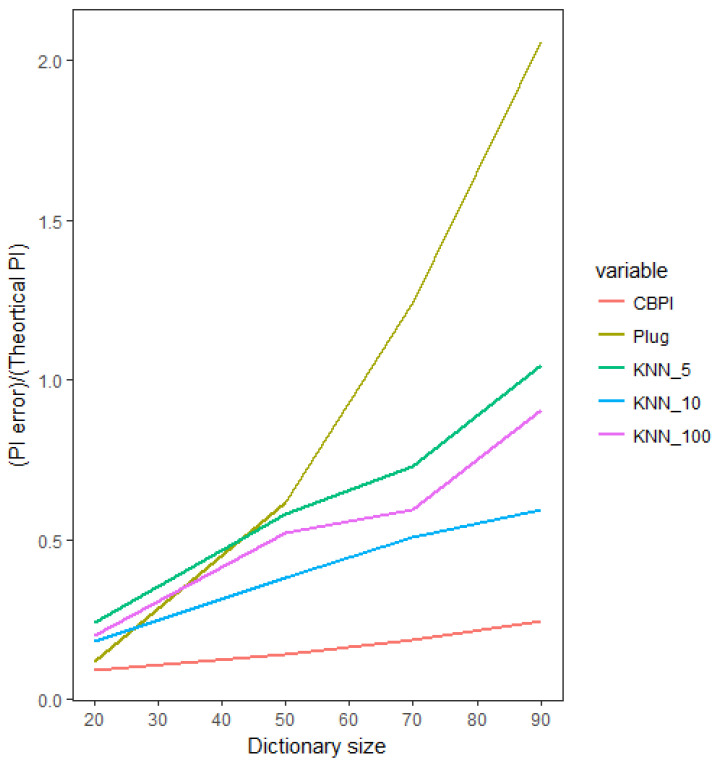
Estimated PI as a function of alphabet size (hence, increasing sparsity). Comparing the CBPI and benchmark methods’ relative estimation error with respect to the true (theoretical) value of the PI.

We now investigate the estimation error, as a function of the sparsity of the significant patterns in the data. In the following setup, we have an input time series with an increasing size of alphabet and a binary output time series. In every run, we chose two symbols to be the informative symbols. The probability of symbols in the output time series, conditioned on the first informative symbol in the input time series, is [0.1, 0.9] for “0” and “1” output symbols, respectively. Similarly, the conditioned probability of symbols in the output time series conditioned on the second informative symbol is [0.9,0.1]. All the other conditioned probabilities are equal to [0.5,0.5] (see Table 4 for the key parameters of this simulation study). This setup allowed us to compute the true theoretical PI value and compare it to the numerically-calculated results. In Figure 3, we compare the CBPI against the best benchmark method from the previous setup, namely the K-NN with various values of K, as well as against the commonly-used plug-in method. In the small number of symbols (low sparsity), the estimation error of the CBPI and the plug-in methods were relatively equivalent (although it is worth mentioning that even in a non-sparse situation with a small alphabet, the CBPI resulted in a smaller PI error), whereas the K-NN error based on all K values was higher. As the alphabet size increased (hence, increasing the sequence sparsity condition), the CBPI resulted in a moderate increase in the estimation error, while all the other methods exhibited a much higher sensitivity to the size of alphabet, resulting in the plug-in methods being the most sensitive to such an increase with the largest error in situations of large sparsity.

### 5.2. The CBPI Algorithm: Example of Real Stocks’ Price Data

Stock market time series analysis is an example of a real-world application of the CBPI algorithm. In this case, the SPI condition is a reasonable assumption because of market efficiency [15]. That is, in an efficient market only a few historical patterns or contexts exist that can be used for predictions, while most of these patterns are insignificant [15]. The dataset comprises minute-by-minute time series of stock prices of eight large banks in the U.S. for the period of January 2008–2010, which because of the banking crisis within these years, has a potential of nonzero $\hat{PI}$ in between banks [16]. The length of the time series was 196,949 minutes; to which, a distributed I/O CT algorithm was implemented. The list of banks and frequency statistics of their stock price changes are shown in Table 5.

**Figure 4:**
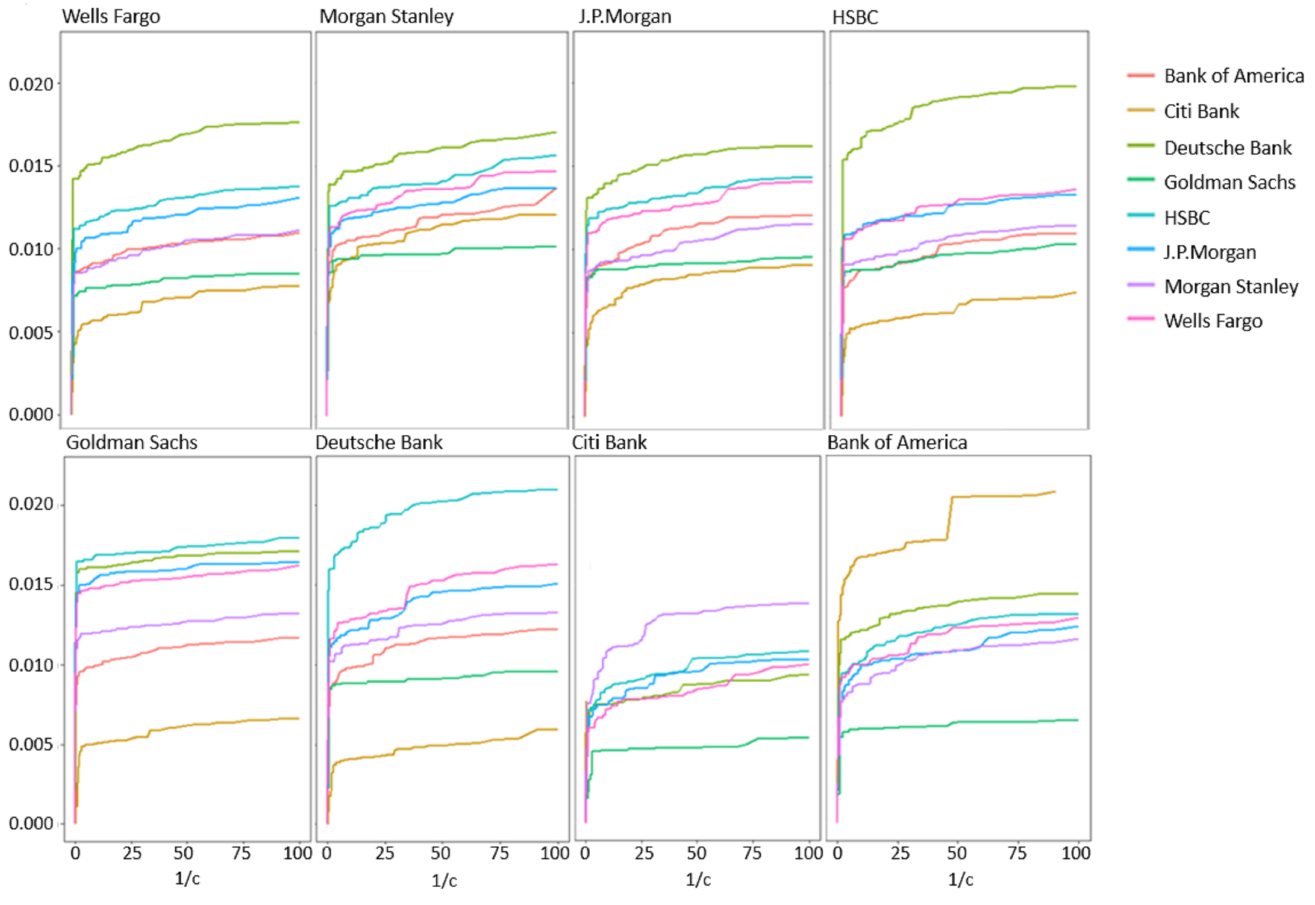
Estimated PI of eight large banks in the U.S. stock market, calculated as a function of the inverse of the pruning constant c. Shuffled input time series showed maximum PI values of ≈$5\times 10^{-5}$.

**Figure 5:**
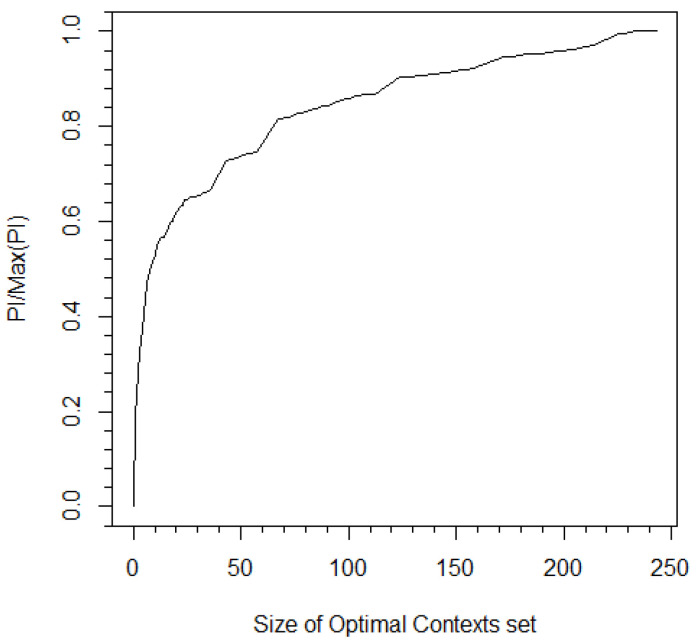
The ratio between PI and the entire I/O CT vs. the size of S. In this case, the PI was calculated between Bank of America and Citi Bank. The pruning constant obtained from the CBPI algorithm corresponds to |S|=10 and a 50 percent ratio of the measured PI to maximum PI.

Stock prices were discretized to +1, 0, and −1 for positive, zero, and negative changes, respectively, relative to the price of the previous minute. For each bank, the PI was obtained by implementing the algorithm of Section 4 for various values of 1/c (see Figure 4). All graphs exhibited a similar behavior of a phase where uninformative sequences were removed followed by a steep drop in PI after crossing a certain pruning constant threshold that corresponded to pruning of sequences from S (BOA had an earlier small steep drop that was followed by an additional plateau). The pruning constant obtained from the CBPI algorithm ranged between 0.13 and 1.33, where more informative input/output pairs were related to a higher pruning constant. These pruning constant values corresponded to filtering approximately 96 percent of sequences while decreasing the $\hat{PI}$ to approximately 50 percent of its maximum value (e.g., see Figure 5).

Using the descriptive power of the CBPI algorithm, hierarchical analysis can be obtained. For example, at the higher level, a geographic orientation can be identified when looking at Figure 4. The estimated PI between the European banks HSBC and DB in both directions was higher than the estimated PI between these banks and the American banks. Another high level observation was related to the total estimated PI (i.e., the sum of all pair-wise $\hat{PI}$ values to all the other banks), which is shown in Table 6. Although these values were relatively close to each other, there was a noticeable difference between the estimated PI value of DB, with the highest total PI value down to Goldman Sachs (GS) with the lowest one. The low value of GS can be related to the fact that it was not influenced by the financial crisis as the other banks; hence, its impact on stock prices’ changes was relatively small. On the other hand, Deutsche Bank’s (DB) total PI value might be surprising due to the fact that it is a European-based bank. Nevertheless, there are some reports that support this result by placing DB as one of the most influential banks world-wide (e.g., see the International Monetary Fund (IMF) technical note from 2016 on: Stress Testing the Banking and Insurance Sectors on Germany).

Moving to lower hierarchies of the interactions, the conditional probabilities of the output sequences given the contexts in S differed from the marginal distribution of the output in the probabilities of each symbol, but the symmetry between −1 and +1 was relatively preserved. For example, see the contexts obtained with the I/O CT of DB to HSBC in the context tree in Figure 1 and Table 1. Therefore, for trading purposes, additional information is needed. By Looking at the tree structure, insightful patterns can be extracted, e.g., it can be seen that along the symmetry axis of the tree (all the optimal contexts that have only zeros), the probability that the price will stay fixed is increasing with the context length, while in the case of more than five minutes with no movement in the stock price, the most probable guess is a zero change with a relatively high probability. Another informative insight that can be drawn is that there are no contexts that contain consecutive change, such as 1>1, 1>−1, −1>1, or −1>−1.

## 6. Discussion

### 6.1. General Comments on the Pruning Constant

**Figure 6:**
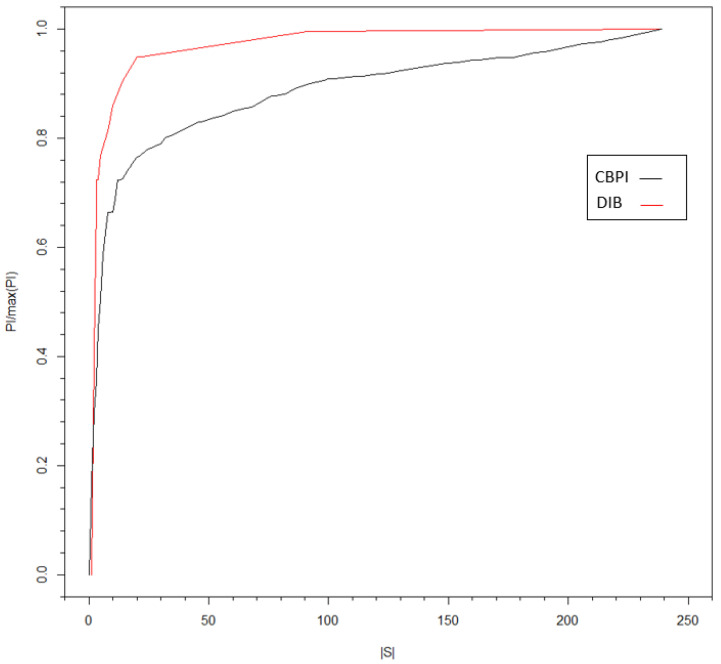
PI rate (with respect to the maximum PI value) versus the size of S, the set of optimal contexts. Both the clusters of the I/O CT and DIB algorithms are shown, respectively, based on the input time series of HSBC and the output time series of DB.

Although the CBPI suggests a justification for the pruning constant value depending on the characteristics of the data, some general principles may apply. The most intuitive one is related to the amount of PI that the optimal contexts hold. When this information is much higher with respect to the uninformative contexts, a higher pruning constant should be expected with an improved separation between the two types of contexts. On the opposite case, if there is a reason to believe that the contribution of the informative sequences is relatively small (as in the bank dataset use-case example), one should consider a more sensitive iterative procedure in the second stage of the CBPI algorithm to avoid the pruning of relatively important sequences. Another principle is drawn from the fact the almost any MI (and PI) estimator has a positive bias in finite data (e.g., see [7] and the references therein). This implies that in smaller datasets, a higher pruning constant is expected (while setting accordingly the ϵ threshold as explained in Section 4). This principle also applies to the alphabet size where a larger alphabet leads to a larger bias. In addition to the above, a typical behavior, across entirely different datasets, can be observed in Figure 4, Figure 5 and Figure 6, with a “knee” curve that can be identified by the CBPI. This behavior is discussed further in the next subsection.

One additional note should be made on recognizing an SPI condition by analyzing the relevant pruning constant values. In particular, if most of the sequences are pruned by a relatively small pruning constant value, and the chosen pruning constant by the CBPI algorithm is relatively high, then the SPI condition is often satisfied. As a rule of thumb, denoting the pruning constant value that removes at least half of the contexts by $c_{0.5}$ and the pruning constant chosen by the CBPI algorithm by $c_{CBPI}$, then the SPI conditioned is when $\frac{c_{CBPI}}{c_{50}}>>1$.

### 6.2. I/O CT and Deterministic Information Bottleneck

In previous sections, we demonstrated how PI can be estimated more accurately by utilizing the connection between PI and the pruning constant in the I/O CT algorithm. The informative sequences can be viewed as a compressed representation of all possible sequences of the input time series, while the compression level is controlled by the pruning constant. The pruning phase in the algorithm can be considered as a sort of a hard clustering procedure of the input with respect to the output time series, where the informative sequences represent clusters of their descendants. This understanding is the reason and the motivation for exploring the connection between I/O CT and the information bottleneck (IB) [19,44] or, more specifically, deterministic IB (DIB) [18].

The goal of the IB algorithm is to find a solution that minimizes the mutual information between a random variable and its compressed representation while keeping a desired level of mutual information between the compressed representation and a target variable. To describe the IB briefly, we follow the notation in [44] and discuss PI in this context. Later, we refer to DIB [18], which links the IB, the PI, and other arguments in this paper.

Denoting by $S_{t}$ the state of the system at time t, the related DIB’s goal is to find a probabilistic mapping (or soft clustering) $P(S_{t}|{\overset{\leftarrow }{X}}_{\tau_{p}})$ such that the following IB Lagrangian is minimized [44], i.e.,

(11) $$\begin{array}{c} \min_{P(S_{t}|{\overset{\leftarrow }{X}}_{\tau_{p}})\;}L_{IB}=I\left(S_{t};{\overset{\leftarrow }{X}}_{\tau_{p}}\right)-\beta I\left(S_{t};{\vec{X}}_{\tau_{p}}\right). \end{array}$$

Thus, the optimal solution for this Lagrangian is the one that achieves the highest compression rate of $S_{t}$ with respect to past trajectories while maintaining the information between $S_{t}$ and the future trajectories at a level regulated by β. From learning and inference perspectives, the optimal $S_{t}$ is obtained by a predictive model that has the lowest complexity with respect to a defined level of predictive power.

DIB [18] formulates a slightly different rate-distortion function, where PI is replaced by the entropy of the compressed representation to restrict its representational cost, namely:

(12) $$\begin{array}{c} \min_{P(S_{t}|{\overset{\leftarrow }{X}}_{\tau_{p}})\;}L_{DIB}=H\left(S_{t}\right)-\beta I\left(S_{t};{\vec{X}}_{\tau_{p}}\right). \end{array}$$

Such a change in the distortion function leads to a deterministic mapping solution where $P(S_{t}|{\overset{\leftarrow }{X}}_{\tau_{p}})$ can obtain values of zero or one [18].

The connection between DIB and I/O CT is obtained through the extraction of the PI introduced in Section 4. We propose to consider I/O CT as a solution of the DIB objective function, such that:

(13) $$\begin{array}{c} \min_{P(S|{\overset{\leftarrow }{X}}_{\tau_{p}})\;}L_{CT}=H\left(S\right)-\frac{1}{c}I\left(S;{\vec{X}}_{\tau_{f}}\right), \end{array}$$

where S, the optimal contexts set, and $\frac{1}{c}$ replace $S_{t}$ and β in Equation (12), respectively. Note that the I/O CT algorithm is asymptotically optimal in its tree source representation [9]. Therefore, for a tree source, the optimal solution obtained from Equation (13) will be equal to the DIB solution. In the general case, optimality is not guaranteed, although similar behavior of I/O CT and DIB is observed, as demonstrated in Figure 6. In this figure, the $\hat{PI}$ was estimated using I/O CT as explained in Section 4 and with the DIB algorithms with different compression levels (controlled by c and β, respectively). Following the DIB approach, the algorithm was implemented on the full I/O CT (with a pruning constant value of zero), to obtain clusters of leaves with similar conditional probabilities. The advantage of such a clustering method over the one based on I/O CT is that it is not limited to the tree structure. Nodes from different parents can be lumped together. However, when the number of contexts in the full tree is large, I/O CT offers a practical implementation of the compression of one time series with respect to another. In addition, the I/O CT algorithm provides a holistic process starting from context parsing to conditional probabilities’ assignment. One can think of a hybrid model where the full I/O CT tree is clustered by DIB. In case of a large number of contexts, the pruning phase can be followed by DIB clustering. These ideas and directions should be further investigated in future research.

## 7. Conclusions

This paper shows how to measure accurately the predictive information (PI) from a given input/output context tree (I/O CT). Using that, we demonstrate how the pruning constant parameter of the I/O CT algorithm can be calibrated in a way that separates informative versus uninformative sequences. This approach constitutes the CBPI algorithm for PI estimation. We used sparse predictive information (SPI) simulated data with known theoretical PI values to benchmark the CBPI algorithm against other common PI estimation methods. This comparison shows the advantages of the CBPI algorithm over the benchmark methods under SPI conditions. The CBPI algorithm was also implemented on real stock prices’ data to show the SPI effect between pairs of real-world time series. It was demonstrated how the CBPI algorithm can be used for in-depth analyses of informative interactions. In the last section, we first discussed some general characteristics of the pruning constant. We then considered an analogy between the CBPI algorithm and the known deterministic information bottleneck (DIB) approach. The rate-distortion optimization problem of the I/O CT algorithm was introduced. We compared the implementations of I/O CT and the DIB on stock prices’ data and demonstrated the difference between the two methods when one time series was compressed with respect to another. Finally, we suggest to explore the concept of a hybrid implementation scheme that includes the use of I/O CT during the first stage, followed by a DIB compression phase to obtain a model with the same bias level, but with a lower variance.

## Figures and Tables

**Table 1:** Optimal contexts of the I/O CT of HSBC to Deutsche Bank as obtained by the CBPI algorithm (see Section 5).

Optimal Context	Context Probability	Conditional Probabilities
root	-	(0.42, 0.16, 0.42)
-1	0.369	(0.45, 0.15, 0.40)
0	0.111	(0.40, 0.20, 0.40)
1	0.370	(0.40, 0.15, 0.45)
-1 0	0.055	(0.43, 0.20, 0.37)
1 0	0.058	(0.37, 0.20, 0.43)
0 0	0.011	(0.37, 0.27, 0.36)
0 0 1	0.011	(0.36, 0.25, 0.39)
0 0 0 -1	0.003	(0.35, 0.30, 0.35)
0 0 0 1	0.003	(0.33, 0.30, 0.37)
0 0 0 0	0.002	(0.32, 0.33, 0.35)
0 0 0 0 1	0.002	(0.36, 0.34, 0.30)
0 0 0 0 0	0.005	(0.06, 0.87, 0.07)

**Table 2:** Key features of the simulation setup. The values in the square brackets are the minimum and maximum that a feature can obtain, where in every run, a value is randomly chosen within these limits. As y is binary, P(y=0|s)=1−P(y=1|S).

Feature	Values
|S|, the optimal context set size	[1, 20]
The length of every context in S	[1, 3]
P(y=1|S)	[0.7, 1]
X alphabet size	10
Y alphabet size	2
Time series length	5000
No. of simulation runs	300

**Table 3:** Comparison of the CBPI algorithm and benchmark methods in a random setup of SPI. The second column from the left shows the average absolute difference error of the estimated PI with respect to the true value. The third column shows the relative mean absolute error with respect to the mean true value. The benchmark methods were calculated using the following R packages: infotheo (Plug-in, MM, Shrink, Sg) [48], entropy(BCMI) [49], and parmigene(K-NN) [50]. In the BCMI method, a parameter of the Dirichlet prior should be chosen. From commonly-used values [49], we chose it to be 0.5, which had the lowest estimation error in this method.

Method	Average Absolute Error (Bits)	Error as a Percentage of the Mean True PI
CPBI	0.005	22%
Plug-in	0.048	200%
MM	0.021	88%
Shrink	0.034	143%
Sg	0.121	510%
BCMI	0.023	100%
K-NN:5	0.031	130%
K-NN:10	0.020	85%
K-NN:50	0.017	70%
K-NN:100	0.020	83%

**Table 4:** Key features of the sparse analysis simulation setup.

Feature	Values
|S|, the optimal context set size	2
The length of every context in S	1
X alphabet size	[20, 50, 70, 90]
Y alphabet size	2
Time series length	5000
No. of simulation runs per each alphabet size	100

**Table 5:** Minute-by-minute stock prices’ change direction for eight large banks in the U.S.: “−1” implies a negative price change; “0” implies no price change; and “1” implies a positive price change. The analyzed banks are: BOA, Bank of America; C, Citi Bank; DB, Deutsche Bank; GS, Goldman Sachs; HSBC; JPM, JP Morgan Chase; MS, Morgan Stanley; WFC, Wells Fargo.

	BOA	C	DB	GS	HSBC	JPM	MS	WFC
−1	81,043 (41%)	68,587 (35%)	81,855 (42%)	94,709 (48%)	83,538 (42%)	87,953 (45%)	86,969 (44%)	84,744 (43%)
0	35,837 (18%)	60,885 (31%)	33,242 (17%)	7235 (4%)	29,290 (15%)	21,149 (10%)	23,541 (12%)	27,401 (14%)
1	80,069 (41%)	67,477 (34%)	81,852 (41%)	95,005 (48%)	83,821 (43%)	87,847 (45%)	86,439 (44%)	84,804 (43%)
Total	196,949 (100%)	196,949 (100%)	196,949 (100%)	196,949 (100%)	196,949 (100%)	196,949 (100%)	196,949 (100%)	196,949 (100%)

**Table 6:** Sum of PI values calculated by applying the CPBI algorithm for each bank with respect to the other banks.

	Total PI (Bits)
GS	0.07
C	0.08
MS	0.09
JPM	0.1
WF	0.1
BOA	0.11
HSBC	0.11
DB	0.12

## Abbreviations

The following abbreviations are used in this manuscript:

c	The context tree pruning constant
CBPI	Context-based predictive information
CT	Context tree
DIB	Deterministic information bottleneck
I/O CT	Input/output context tree
IB	Information bottleneck
MI	Mutual information
PI	Predictive information
S	Optimal context set
SPI	Sparse predictive information

## Appendix A

This Appendix describes the main steps of the context tree algorithm. In the last part, a walk through example of the first stage of the algorithm of the tree construction is given [10].

Given a time series $x^{1}\ldots x^{T}$ of length *T* where every symbol in the sequence is one of d symbols, *x* ∈ {1, 2, …, *d*} and *t* ∈ {1, …, *T*}, do the following steps:

1. **Counter tree construction:** From the given sequence, construct recursively a counter tree, where every node represents a context s of symbols in the series and the counts of symbols that comes after this context n(x|s). The tree starts from the root node, that is the counts of every symbol in the time series.
2. **First pruning step:** denoting the length of a context as l, prune all contexts such that $l\geq \frac{log(T+1)}{log(d)}$.
3. **Second pruning step:** The distribution difference between a node and its parent node is measured by $D_{K}L$ and should be higher than a threshold governed by the pruning constant c:
  $$\begin{array}{c} \Delta_{N}\left(sb\right)=\sum_{x\in X}n\left(x|sb\right)log\frac{p(x|sb)}{p(x|s)}\geq c\left(d+1\right)log\left(T+1\right);b\in X \end{array}$$
4. **Finding optimal contexts**: The contexts set S is comprised of contexts that are leafs or partial leafs that satisfy the following condition, which identifies contexts that are not contained in their unpruned children nodes:
  $$\begin{array}{c} S=\{s:\sum_{x\in X}\left[n\left(x|s\right)-\sum_{b\in X}n\left(x|sb\right)>0\right]\} \end{array}$$
5. **Estimation of the CT probability parameters:** First, the probabilities of the optimal contexts P(s);(s∈S) are estimated by the equation below where the numerator is the number of symbols that belong to a context s and not to another context:
  $$\begin{array}{c} \frac{\sum_{x\in X}\left[n\left(x|s\right)-\sum_{b\in X}n\left(x|sb\right)\right]}{\sum_{s\in S}\sum_{x\in X}[n\left(x|s\right)-\sum_{b\in X}n\left(x|sb\right]} \end{array}$$
  Second, the conditional probability of symbols given its context is obtained by the frequency of a symbol given its context divided by the sum of the symbols that belong to this context:
  $$\begin{array}{c} P\left(x|s\right)=\frac{n\left(x|s\right)-\sum_{b\in X}n\left(x|sb\right)}{n(s)} \end{array}$$

Step 1 of the tree construction is somewhat less intuitive than the rest of the algorithm. Giving a short toy example for this stage will clarify its logic. Consider two random variables X and Y, represented by two time series with a length of five. X realizations are 11000, and Y realizations are 00111 (t = 1 is on the left). The tree construction will now be demonstrated on Table A1, remembering that the three edges are contexts from x and the nodes represent the counters of the symbols following each context.

**Table A1:** Context tree construction example following the first stage of the context tree algorithm.

Time	Context Tree	Explanation
**t = 1:**		Root node with counter [1,0] is added to the tree, because 0 is the first symbol in Y.
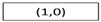
**t = 2:**		Add the context node 1 to the tree, and increment the one counter by 1. Update the counter of the root node.
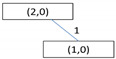
**t = 3:**		Add the context node 11 to the tree and increment the one counter by 1. Update the counters of all its predecessor nodes.
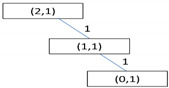
**t = 4:**		Add the context nodes 0; 01; 011 and increment their one counters by 1. Update the root counter.
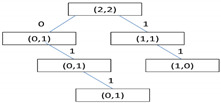
**t = 5:**		Add the context nodes 00; 001; 0011 and increment their one counter and their predecessors’ counter by 1.
